# How EFL Teachers Engage Students: A Multimodal Analysis of Pedagogic Discourse During Classroom Lead-Ins

**DOI:** 10.3389/fpsyg.2021.793495

**Published:** 2021-12-23

**Authors:** Yongli Qin, Ping Wang

**Affiliations:** ^1^School of Foreign Languages, Jiangsu University of Science and Technology, Zhenjiang, China; ^2^Graduate School, Xi’an International Studies University, Xi’an, China

**Keywords:** engagement, EFL teachers, pedagogical discourse, multimodal, classroom lead-in

## Abstract

Classroom lead-in is the initial stage for motivating students to become engaged in-class interaction. However, little research, to our knowledge, has analyzed the role of teachers’ multimodal competence reflected through their multimodal pedagogic discourse in the realization of the ultimate goals of classroom lead-ins. Based on the data collected from a teaching contest in China, this paper explores how two-winner teachers utilize their multimodal ensembles of communicative modes to engage students during classroom lead-ins. The analysis shows that different communicative modes construct the higher-level action of lead-in, and they are orchestrated into multimodal ensembles for the specific function of each lead-in move. The findings indicate that EFL teachers’ high multimodal competence plays a decisive role in performing classroom lead-ins, and different lead-ins strategies influence the different orchestration of communicative modes. In constructing multimodal pedagogic discourse, teachers build up their professional image and display their personal charm as well. Future research for multimodal discourse analysis and pedagogic research is suggested in the paper.

## Introduction

Multimodality has become a hot topic in language education research (Kress, 2010; Rowsell and Collier, 2017; Canagarajah, 2018; Peng, 2019). As in all educational contexts, teaching English as a foreign language (EFL) in Chinese universities is a multimodal experience that happens through the orchestration of spoken language and an array of other communicative modes, such as gesture, gaze, and facial expression (Peng, 2019). However, EFL classrooms are different from others in that EFL teachers are advanced EFL learners themselves, and students mostly learn and use English in class. Therefore, in order to maximize learning, teachers often make great efforts to motivate students to participate in class interaction (Peng et al., 2017; Peng, 2019). EFL teachers’ multimodal competence – the ability to select and combine different communicative modes besides spoken language to complement or support their use of English as the medium of instruction for various teaching purposes – appears to be particularly important as it contributes to effective learning (Morell, 2018). In the context of the EFL classrooms, effective pedagogy often starts with a good lead-in, a technique utilized by teachers to prepare students to learn and involve them in-class participation (Turney, 1975; Arendas, 1998; Gardner and Miller, 1998; Slavin, 2004).

Engagement is one of the hottest research topics in the field of educational psychology. Research shows that a plethora of benefits occur when students are engaged in their learning (Sinatra et al., 2015, p. 1). However, to the best of our knowledge, the study of the relations between teachers’ multimodal competence and engagement is scanty. Seizing the gap, this study intends to explore how teachers’ multimodal competence contributes to the realization of the ultimate goal of classroom lead-in: to engage students. The ultimate aim of this study is twofold.

On the one hand, we intend to expand the knowledge of multimodal research by analyzing classroom discourse during the lead-in from a multimodal perspective while considering that all semiotic resources play a role in meaning-making. In this sense, we are interested in exploring what semiotic modes are used and how they are combined to achieve communicative purposes. On the other hand, we aim to reveal the significance of teachers’ multimodal competence in engaging students, intending to arouse teachers’ awareness of multimodal competence and learn to make appropriate use of it when they design classroom lead-ins.

## Literature Review

### Classroom Lead-Ins

Classroom lead-in is the first pedagogic procedure used to “awaken” students in the first 3–5 min and motivate them to learn. Arendas (1998: 240) delimits lead-in as “a technique by teachers at the beginning of a new lesson or when presenting new knowledge to prepare students to learn and establish a communicative link between the learners and the information about to be presented.” Turney (1975) believes that it is essential to attract students’ attention and cultivate their desire for learning at the beginning of teaching. Since a good beginning is half done, a good classroom lead-in is decisive for successful classroom teaching (Liu and He, 2020). Many researchers have reached agreements on the significant role of classroom lead-in in effective teaching that the lead-in is a crucial link of classroom teaching (e.g., Gardner and Miller, 1998; Liu, 2004; Slavin, 2004; Fu, 2005). Turney (1975) categorizes the multiple functions of classroom lead-in into five types: gaining attention, arousing motivation, setting up a teaching target, structuring, and making links. These functions would enable students to get psychological preparation for the new class. Cooper (1992) maintains that the ultimate goal of the lead-in is to motivate students to participate in class activities or engage students.

Concerned with its significance to effective teaching, many researchers have proposed guidelines and strategies to make a good lead-in. For example, Gower and Walters (1983) suggest that lead-in should be socializing and the lead-in topic should be related to students’ daily lives; Slavin (2004) requires that teachers attract students’ attention and provoke their curiosity. Liu and He (2020) summarize the previous research and state that a good lead-in should be interesting, relevant, student-centered, brief and authentic, and close to students’ life. They also suggest a range of lead-in strategies that a teacher can choose depending on their preference: situational lead-in, multi-media lead-in, dialogue lead-in, revision lead-in, question lead-in, hot topic lead-in, and storytelling lead-in. These guidelines and strategies are conducive for teachers to design a lead-in during the lesson-planning stage.

In the sense of genre analysis, classroom lead-in is a specific genre as it is a communicative event shared by the teacher and students in a classroom setting (a “discourse community”) and with a common goal or the communicative purpose (Swales, 1990, p. 58). Thus, it can be analyzed in terms of its structure and communicative purposes. The discourse structure is described as a sequence of “moves,” where each move represents a stretch of discourse serving a particular communicative function. Therefore, following a top-down approach by identifying functions or communicative purposes that classroom discourse can serve during the lead-in, we can segment the lead-in into different moves and note the type of each move. Chinese Scholar Zhang (2015, p. 169–172) puts forward a comprehensive lecture structure consisting of 16 moves, based on a sample of six classroom teaching videos of national excellent EFL courses for undergraduates. Following Zhang’s (2015) classification of EFL classroom structure and making necessary modifications, we propose that an EFL classroom lead-in includes four moves (see Table 1) in line with some of Turney’s (1975) identification of lead-in functions: it begins with a move that the teacher greets with students, followed by a move that clarifies the teaching plan, introduces the upcoming topic, and declares the shift to the next section.

**Table 1:** Moves of EFL class lead-in.

S. no.	Moves	Functions
1.	Greeting	Getting attention: declaring the start of lesson and establishing/maintaining an interpersonal relationship with students
2.	Introducing teaching plan	Setting up teaching target and structuring: Getting students to know the teaching/learning goals and how to achieve them
3.	Lead-in activities	Arousing motivation: Familiarizing students with the topic and get them prepared according to different teaching contents for features
4.	Closing the lead-in	Making links: making links between lead-in and the following section and directing students to the next teaching procedure

The sequence of move 2 and move 3 is interchangeable.

### Multimodal Pedagogical Discourse

Multimodal research first appeared as early as in the latter half of the 1990s in the studies of meaning-making, conveying, and receiving (Jewitt et al., 2016). As a new paradigm for discourse analysis, multimodal discourse analysis extends the study of language itself to the study of language combined with other semiotic resources (such as images, gestures, actions, and music) in the meaning-making process (O’Halloran, 2004). The multimodal pedagogic analysis primarily draws on a social semiotic perspective (Kress, 2010) or on systematic functional multimodal analysis (SF-MDA) to explore the multimodal meaning-making of pedagogic discourse in different educational contexts and disciplines (Morell, 2018).

In the literature of multimodal analysis of pedagogic discourse, studies involve educational contexts of different levels, including primary and secondary schools (e.g., Kress et al., 2005; Jewitt, 2008), high schools (e.g., Young and Nguyen, 2002), and higher education seen in the special issue of *System* in 2018 (e.g., Coccetta, 2018; Franceschi, 2018; Morell, 2018), and even in 3-D virtual worlds for language teaching and learning (Tan et al., 2016). They have explored multimodal discourse of various disciplines, like science (Kress et al., 2001; Young and Nguyen, 2002; Tang, 2013), mathematics (O’Halloran, 2004, 2005, 2010), and English (Jewitt, 2002; Kress et al., 2005; Jewitt, 2008). Some studies have focused on specific semiotic modes for meaning-making in classrooms – for example, gestures (Bezemer, 2014; Cao and Chen, 2017; Lim, 2017), classroom space (Lim et al., 2012), gestures and facial expressions (Sueyoshi and Hardison, 2005), or language and images (Lotherington et al., 2019). Some have explored the effects of multimodal pedagogic discourse on classroom teaching efficiency (Sueyoshi and Hardison, 2005; Moreno and Mayer, 2007; Guichon and McLornan, 2008; Morell, 2018).

These studies challenge the traditional view that teaching and learning are primarily accomplished through pedagogic language. The multimodal approach to classroom discourse reveals that the pedagogic language is not the only mode to construct or display knowledge. It is the process where several semiotic resources work together during social interaction within the classroom (Lim, 2021). Lim (2021) also observes that the teachers’ use of gestures, the classroom spaces they occupy, and the movements they make and the tools they use work together with language as a multimodal ensemble of meanings. This is especially crucial for language learning and teaching because it is a process mediated in interaction with language teachers and students, where the medium of learning is often also the content (i.e., language; Lantolf, 2000; Lamy and Hampel, 2007). Hence, “it is now impossible to make sense of texts, even of their linguistic parts alone, without having a clear idea of what these other features might be contributing to the meaning of a text” (Kress, 2000, p. 337).

However, the research into multimodal EFL pedagogic discourse and their role in engaging students in class in higher education is less than adequate. A few studies have shown that a teacher’s facial expressions, gestures, and spatial positions are fundamental communicative modes that contribute to EFL learners’ willingness to communicate in English language classrooms (Peng et al., 2017; Peng, 2019). Morell (2018) has illustrated that coordinated use of complementary mode ensembles together with language enables teachers to organize and interpersonally involve students in class activities textually. Morell et al. (2020) uncover that trained English-medium instruction (EMI) lecturers combine semiotic resources (e.g., gaze, gesture, and written language) with their use of verbal discourse to engage students, and the orchestration of communicative modes serve to foster engagement in the classroom. These studies suggest that multimodal classroom pedagogies can promote EFL learners’ willingness to communicate, which results in effective interactive lecturing. However, these are all macro-investigations that examine the teaching process as a whole devoid of delving into one specific teaching procedure, for example, classroom lead-in.

In the context of the EFL classrooms, effective pedagogy often starts with a good lead-in, and a good lead-in involves EFL teachers’ multimodal competence. The lead-in is the initial stage for motivating students to become engaged in-class interaction. Previous research into lead-in highlights its functions and proposes guidelines and approaches on making a good lead-in (e.g., Slavin, 2004; Velandia, 2008). However, previous research seldom analyzes the multimodal process of how teachers implement lead-in to engage students in English classrooms. Up to our knowledge, only a couple of researchers have examined EFL classroom lead-ins, but their studies are mainly from the pragmatic perspective. For example, Qin (2015) explores the EFL teacher’s multimodal pedagogic discourse during the lead-in from the adaption theory; Li and Zhou (2012) investigates the EFL teachers’ multimodal actions in the lens of politeness theory, and they find that inappropriate multimodal behaviors might distract students’ engagement in-class participation. Therefore, to enrich the research into lead-in, the present study intends to reveal how teachers’ multimodal competence contributes to students’ classroom engagement by analyzing lead-ins of two demo classes as an example.

### Multimodal Interaction Analysis

Multimodal interaction analysis (MIA) is a theoretical and methodological framework proposed by Sigrid Norris to analyze a multiplicity of interactions that social actors are simultaneously engaged in. It is a more interactional approach toward multimodality where the focus is on the actions carried out by the social actor (Norris, 2004, 2011). It is initiated by applying the theoretical notion of mediated discourse proposed by Scollon (1998, 2001) and employing visual research methods to discourse analysis (Kress and van Leeuwen, 2001). The theoretical framework of mediated discourse analysis focuses on human action and encourages the integration of nonverbal communicative modes into discourse study. Meanwhile, visual methods promote the analysis of many communicative modes and help researchers investigate the intricate interplay between various modes in communicative events (Norris, 2011, p. 3).

MIA places a considerable emphasis on the notion of context and situated interaction (Jewitt, 2014). Moreover, thus, the social actor becomes central in MIA studies as the actions carried out by the social actor with or through multimodal mediational means are the focus for analysis (Norris, 2004). Therefore, this approach aims to figure out the intricacies of interactions and how social actors behave in specific instances (Norris, 2004). Norris (2004, p. 4) explains, “Multimodal Interaction Analysts set out to understand and describe what is going on in a given interaction.”

MIA shifts attention from representation and communication, the focus of approaches taken by Kress, van Leeuwen, and O’Halloran, to interaction (Jewitt, 2014). In Norris’s (2004, p. 149) view, communication is interaction if “one person conveys a message and another perceives it.” Here, the focus of interaction is expanded, moving away from linguistic interaction to explore how people employ gesture, gaze, posture, movement, space, and objects to mediated interaction in a given context. As a corollary of the focus shifting to interaction, the modal system is no longer a primary concern.

In multimodal interactional analysis, the *mediated action* is the unit of analysis, and it can be further categorized into three layers of actions: *lower-level actions*, *higher-level actions*, and *frozen actions* (Norris, 2004, p. 13; Scollon and Scollon, 2004). Lower-level actions are fluidly performed in interaction and are mediated by an array of communicative modes, including body parts, hands, arms or figures, etc. Moreover, the sum of fluidly performed chains of lower-level actions develops into higher-level actions. Social actors orchestrate a range of multiple modes of communication in interactions to accomplish various higher-level actions simultaneously (Norris, 2011). Frozen actions are higher-level actions frozen in a material object. For example, the objects present in a classroom construct the mode of layout, which gives off messages about the social actor and structure the interaction somehow. According to Norris (2004, 2019, 2020), the higher-level actions are also fluid and develop in real-time, and each higher-level action is bracketed by social openings and closings that are at least in part ritualized.

Jewitt (2014, p. 36) holds that multimodal interaction focuses on the mediate interaction in a given context, that is, how a variety of modes are brought into and constitutive of social interaction. Therefore, the first step to a multimodal interaction analysis is a basic understanding of a multiplicity of communicative modes (Norris, 2004, p. 11), which are essentially systems of representation with rules and regularities attached to them (Kress and Van Leeuwen, 2001). And, since the modes are interdependent upon one another in many different ways and the structure has to be determined through specific analysis with the consideration of occurring environment and context (Norris, 2004), the analyst will then try to investigate how these communicative modes play together to make sense in interaction. Norris (2004) used modal intensity to refer to the intensity, or weight one communicative mode carries in interactions. If a mode takes on primacy in a specific interaction, it takes on high intensity. This can happen to any mode or several jointly interconnected modes. When several modes communicate together in synchrony without one communicative mode taking on particular interactions, the intricate interplay among modes is called *modal complexity*. Modal complexity and modal intensity can also combine. That is, a hierarchically structuring mode is an intense mode that constructs other modes in interaction. It can work with other complexly interconnected modes. The notion of *mode density* indicates the level of attention/awareness that a social actor plays on a particular mode, which is achieved through model intensity or modal complexity or both when constructing a higher-level action. White (2010) has utilized the concept of modal density to analyze the New Zealand Army interactive posters and shows how communications in the age of information overload are more likely to be successful if they find new ways of getting and keeping attention.

Although MIA is “still in its infancy” (Norris, 2014, p. 98), it has recently been applied in various studies. Norris (2004) utilizes the framework of multimodal interaction analysis to illustrate how a teacher utilized an interactive means in a language instruction classroom discourse to shift students’ attention to a new higher-level action. Norris (2011, 2017) has illustrated the efficacy of the framework for constructing a social actor’s social world in everyday interactions, like identity production, complexity, and cultural differences in different nations. Lotherington et al. (2019) analyzed how language and images interact as meaning-making resources in constructing a plurilingual talking book from the multimodal interaction perspective. Pirini is one of the supporters of this approach. In a series of studies, Pirini (2014) explores shared attention/awareness in high school tutoring sessions and develops the notions of *agency* in intersubjectivity in tutoring sessions, among others (Pirini, 2013). Within the field of education, Fernández-Pacheco (2016, 2018) has recently adopted the concept of *higher-level action* to structure a series of vodcasts with the aim to analyzing which multimodal ensembles are more beneficial for students’ comprehension; Bernad-Mechó (2017) uses MIA to examine how topics are introduced through the use of *introducing topic* metadiscourse in a history lecture, and he then explores the complex process of lecture structuring by analyzing actions carried out by the lecturers (Bernad-Mechó and Fortanet-Gómez, 2019).

To sum up, previous research has shown that MIA allows for an in-depth exploration of human interaction with a particular emphasis on the social actor, in which the mediated action becomes the basic unit for analysis. It is believed that the exploration of various interactions based on this perspective undoubtedly will bring new developments to the field of multimodality (Norris, 2014). In the present research, MIA is adopted as a theoretical and methodological framework to scrutinize the mediated actions performed by EFL teachers. This allows for a closer, more interpretative way to reveal how EFL teachers engage students during the lead-ins. These three research questions guide this study.

1. What multimodal communicative modes are selected and utilized by EFL teachers when they construct the higher-level actions of the lead-in?
2. How are communicative modes assembled to realize the communicative purpose of each move of classroom lead-ins?
3. What role does EFL teachers’ multimodal competence play in engaging students during classroom lead-ins?

## Methodology

### Research Settings

This study reports on two case studies of two highly-qualified EFL teachers, the top two winners of a national teaching contest held for college English teachers in 2015. The teaching contest is an annually held event to provide a platform for college English teachers to demonstrate their teaching strategies and skills. The annual competition has attracted EFL teachers from nearly one thousand colleges and universities across the country, and only those who stand out from their provincial contests will have the opportunity to attend the semi-finals, and only the top 10 semi-finals winners will advance to the national final. Thus, in this paper, we take those winner teachers of national finals as highly-qualified English teachers for their profound language skills, excellent teaching performance, and elaborate curriculum design.

Contestant teachers are required to present a 20-min lecture with a complete teaching procedure as a regular classroom does, including the lead-in, presentation, practice, and homework assignments. The demo course, called *Intensive Reading*, is a comprehensive reading course designed for English majors in China, aiming at improving students’ basic skills of reading, listening, speaking, and writing. The topics for demo class are determined by drawing lots 2 weeks ahead of the competition. The contests are held in the same lecture room seated with participant students and expert judges. Furthermore, constant teachers do not know the students in advance, and the students know nothing about what topic will be introduced, either. Hence, it is quite a challenging task for contestant teachers to engage students during the lead-in section. The complete teaching procedure of each contestant is recorded and published nationwide together with winner teachers’ personal reflection on the contest, their PPT, and expert judges’ comments on each contestant’s performance. These resources are also available online for use in pedagogic purposes.

### Participants

Two top winners of the national finals are selected as participants for this study. They are EFL teachers from two different universities located in different provinces in China. For the convenience of reference, one teacher is referred to here as T1 and the other as T2. The demographic information of the two teachers is shown in Table 2. These two teachers are chosen as participants for the following considerations. Firstly, they are the top two winners of the national finals who also happened to be of the opposite sex. Secondly, they taught the same group of students (demographic information is shown in Table 3) with different topics, fully presenting different lead-in strategies during their demo class. T1’s topic is “Life as a Housefather,” and he uses storytelling lead-in to provoke students’ interest in the story of a housefather; and T2’s topic is “The Quest for Convenience” and she adopts question-answer style to motivate students’ participation in the topic discussion. Thirdly, they adopt different lead-in strategies with different time allocation, but both teachers successfully engage students and establish a positive classroom atmosphere for the smooth transition to the reading and appreciation of the given text. Their different ways to introduce the topic of the text are well received by the students (judged from the reaction of students in the video) and highly praised by expert judges as “simple and effective, which aroused students” interest in the topic, and prepared them for the study of the text’ [National Advisory Committee on Teaching English to Majors in Higher Education (NACTEMHE), 2016, p. 30].

**Table 2:** Demographic information of the two teachers.

Background information	T1	T2
Gender	Male	Female
Age	35	36
Yeas of EFL Teaching	14	15
Education	MA	MA
Major	Translation	Literature
Language proficiency	Fluent English speaker	Fluent English speaker
Award rank	1st-prize	2nd-prize
Demo class topic	Life as a House Father	The Quest for convenience
Lead-in strategy	Storytelling	Question-answering
Lead-in time allocation	2'36	3'48
Total time duration of demo class	20’	20’

**Table 3:** Demographic information of the students.

Participant students		
Gender	Male	3
	Female	9
Age	Maximum	19
	Minimum	17
Years of English Learning	Maximum	12
	Minimum	10
Major	English	
Grade	Freshmen	
Familiarity with demo-lecturers	Total strangers	Total strangers

### Data Collection

**Figure 1:**
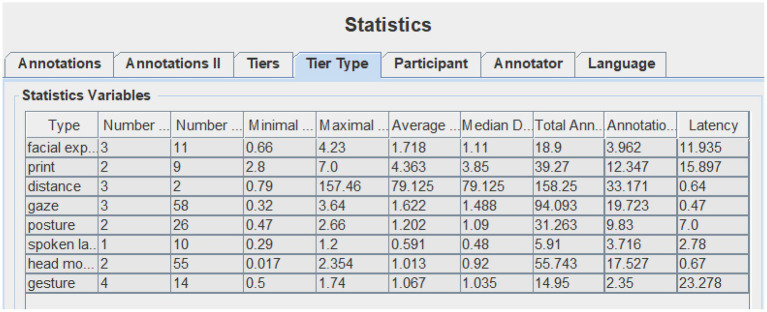
Screenshot of T1’s communicative modes on ELAN statistics.

**Figure 2:**
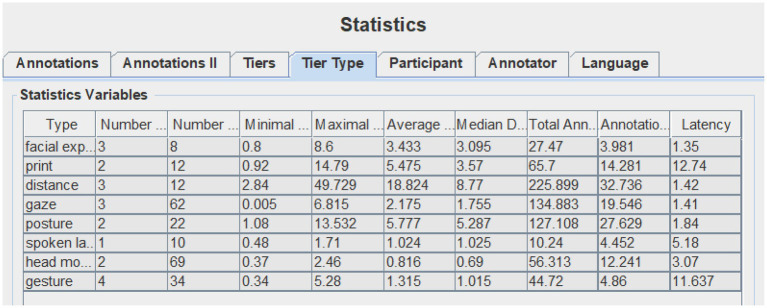
Screenshot of T2’s communicative modes on ELAN statistics.

Data collected for this study include recorded videos of demo class, expert judge’s comments, contestant teachers’ self-reflection on their teaching, and their PPT (available online: https://we.sflep.com/Teaching/PastCompetition.aspx?id=6). For the video clip, this study uses the ELAN multimodal analysis software (EUDICO Linguistic Annotator) to annotate communicative modes (Version 5.8 of this software can be downloaded from the official ELAN website, https:tla.mpi.nl/tools/tla-tools/elan/download/). To annotate the data, the researchers first clipped out the lead-in segments and then watched and observed the two sample-videos for numerous times to note the features of different modes. Based on previous research into multimodal teaching and Norris’ (2004) list of communicative modes for everyday multimodal interactions, we annotate the following modes: spoken language, print, distance, posture, gesture, gaze, head movement, and facial expression. The eight communicative modes were further annotated with ELAN (see Figure 1) based on a coding schema (see Table 4). After the annotation, the ELAN can provide the annotation statistics, including numbers of annotation, average duration, total annotation duration, annotation duration percentage, etc. seen in Figures 1, 2.

**Table 4:** Coding schema of communicative modes.

Communicative modes		Tier and Coding
Language	Spoken language (SL)	Prosody (SLP)
	Print(P)	Writing on the white board (PWWB), PPT slide (PPS)
Distance (D)		Formal distance (DFD), Social distance (DSD), Personal distance (DPD)
Posture (P)		Closed posture (PCP), Open posture (POP)
Gesture (G)		Iconic gesture (GIG), Deictic gesture (GDG), Metaphoric gesture (GMG), Beat gesture (GBG)
Gaze (G)		Gaze at all the students (GGAS), Gaze at one student (GGOS), Gaze at the PPT presentation (GGPP)
Head movement (HM)		Directional shift (HMDS), head beats (HMHB),
Facial expression (FE)		Positive (FEP), Negative (FEN)

### Analytical Procedures

The researchers follow the procedures suggested by Norris (2004, 2019, 2020) to analyze two EFL teachers’ multimodal pedagogic discourse during classroom lead-ins. According to Norris (2004, 2019, 2020), the first step to a multimodal interaction analysis is to understand an array of communicative *modes*. The ELAN annotation of communicative modes helps us achieve the first step already. After that, we will investigate how modes play together in interaction. Here, the classroom lead-in is seen as a higher-level action, and the four moves are four lower-level actions. We will analyze the interplay of communicative modes at each move level and assemble them into patterns according to their modal intensity. Last of all, based on the findings of the first two steps, we will discuss the role of teachers’ multimodal competence in engaging students during classroom lead-ins.

## Results and Analysis

This study first presents the descriptive data of communicative modes used by two teachers and then illustrates how communicative modes work together to realize the communicative purpose of lead-in.

### Communicative Modes in Pedagogical Discourse

The annotation statistics provided by ELAN (see Table 5) show that both teachers made full use of eight communicative modes to construct a multimodal pedagogic discourse during the classroom lead-ins. There is no significant difference between them in terms of multimodal behaviors in general (*F* = 0.80311486, value of *p* = 0.399931623, Fcrit = 5.591447851). However, the statistics indicate that two teachers differ significantly in their performance of each specific communicative mode (*F* = 8.596998143, value of *p* = 0.005510677, Fcrit = 3.78704354). The variance value of each communicative mode indicates the similarity or difference in teachers’ multimodal behaviors. The bigger the variance value, the larger is the difference between them. From the values, we can find that among the modes utilized by both teachers, those that help to build interpersonal relationships are preferred, like facial expressions, gaze, and distance, while the other modes that are associated with lead-in strategies take on different application frequencies, like gesture, head movement, and posture. Table 6 presents the detailed information of each communicative mode utilized by two teachers. The following is the use of communicative modes between two participants during classroom lead-ins.

**Table 5:** Communicative modes in the classroom lead-ins of two demo-lectures.

	T1				T2				
Mode	NoA	AD	TAD	ADP	NoA	AD	TAD	ADP	Variance
Spoken language	10	0.591	5.91	3.716	10	1.024	10.24	4.452	0.270848
print	9	4.363	39.27	12.347	12	5.475	65.7	14.281	1.870178
Distance	2	79.125	158.25	33.171	12	18.824	225.999	32.736	0.0946125
Gaze	58	1.622	94.093	19.723	62	2.175	134.883	19.546	0.0156645
Posture	26	1.202	31.263	9.83	22	5.777	127.108	27.629	158.4022005
Head movement	55	1.013	55.743	17.527	69	0.816	56.313	12.241	13.970898
Gesture	14	1.067	14.95	2.35	34	1.315	44.72	4.86	3.15005
Facial expression	11	1.718	18.9	5.942	8	3.433	27.47	5.971	0.0004205

NoA = Number of Annotations, AD = Annotation Duration, TAD = Total Annotation Duration, ADP = Annotation Duration Percentage.

**Table 6:** Annotation statistics of each type of communicative mode.

Mode	Types	T1			T2		
		NoA	TAD	ADP	NoA	TAD	ADP
Facial expression	FEP	5	10.86	6.829	7	26.0	11.303
	FEN	6	8.04	5.056	1	1.47	0.639
Gaze	GGAS	51	85.653	53.862	34	74.508	32.391
	GGOS	0	0	0	11	23.275	10.119
Distance	DFD	1	0.79	0.497	3	91.28	39.683
	DSD	1	157.46	99.018	5	121.469	52.807
	DPED	0	0	0	4	13.15	5.717
Spoken language	SLP	10	5.91	3.716	10	10.24	4.452
Print	PPS	9	39.27	24.684	4	40.32	17.529
	PWWB	0	0	0	8	25.38	11.034
Gesture	GIG	0	0	0	1	6.3	2.739
	GDG	11	12.5	7.86	13	11.71	5.091
	GMG	0	0	0	11	12.19	5.299
	GBG	3	2.45	1.541	9	15.54	6.756
Head movement	HMDS	34	38.991	24.519	60	50.393	21.908
	HMHB	21	16.752	10.534	9	5.92	2.574
Posture	PCP	22	28.493	17.917	13	51.119	22.223
	POP	4	2.77	1.742	10	76.266	33.156

NoA = Number of Annotations, TAD = Total Annotation Duration, ADP = Annotation Duration Percentage.

#### Language

In an English as a foreign language classroom, a target language is a tool for teachers to organize classroom teaching and an important source of language input. This study represents language mode by spoken language, print mode of PPT slides, and blackboard writing. Spoken language is the primary mode with the most vigorous intensity among all the communicative modes in the language classroom setting. Spoken language is analyzed in terms of prosodic features where stress and pause are annotated. The statistics of ELAN annotation show that both teachers have the same number of prosodic features but differ in annotation duration. There is no significant difference, in any case. As for pause, T1 seemed to use it as a technique for storytelling lead-in. For instance, after his comment with a tag question, “That sounds perfect, right?,” T1 paused to check students’ reactions. This is also the case of T2. After she raised a question, she paused to observe the students’ responses. Since T2’s question-answer lead-in needs more students’ participation, she also used more prosodic features, like high pitch or stress, when asking or responding to students’ answers. Besides spoken language, both teachers depend more on a computer-mediated PPT screen to present information, but T2 also uses the whiteboard to present information, which makes up for the shortage of PPT slides. One expert judge spoke highly of T2’s use of the whiteboard, saying that it is a “smart and effective way to catch students” attention on the keywords which presuppose the theme of the text’ [National Advisory Committee on Teaching English to Majors in Higher Education (NACTEMHE), 2016, p. 12].

#### Distance

**Figure 3:**
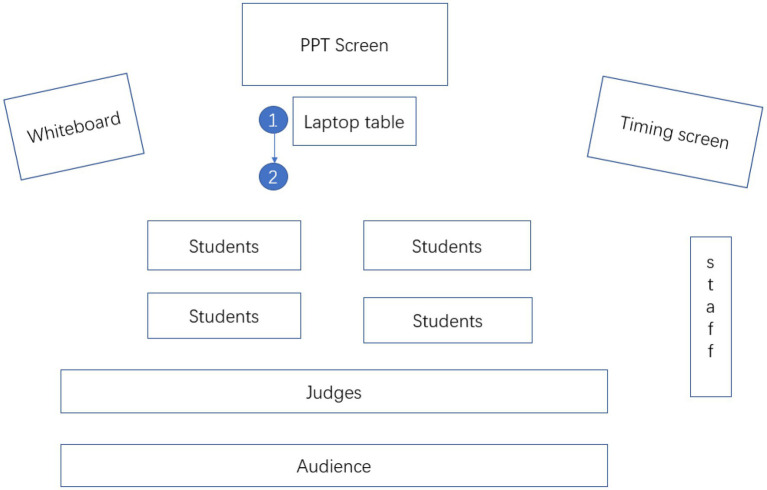
T1’s distance shift (the number show the sequence of movement).

**Figure 4:**
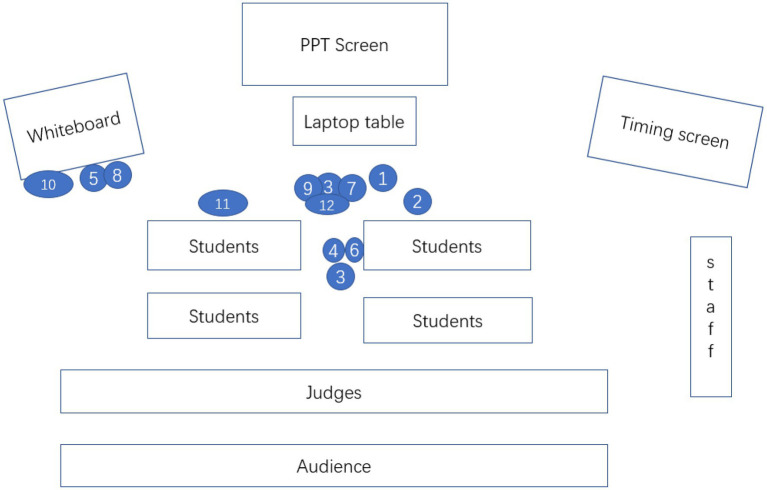
T2’s shifts of distance (the numbers show the sequence of movement).

The distance that a teacher takes up for students allows us to gain insight into social relationships. Based on Hall’s (1966) work on proxemics, we examined three types of distance as the close distance is rare in the formal classroom: formal distance, social distance, and emotional distance, which corresponds to the three types of space in the classroom proposed by Lim et al. (2012): authoritative space, interactional space, and personal space. In the study, the three distances are redefined depending on the specific layout of the furniture in the classroom. Formal distance refers to the position where the teacher stands around the laptop table or near the whiteboard. The social distance is where the teacher stands in or near the passageways between students’ desks, and the personal distance refers to the position where the teacher intentionally stands beside one student. The result in Table 6 shows that both teachers prefer to keep a social distance with students as it both highlights the teachers’ authoritative position in the class and facilitates interaction with the entire student. To be specific, T1 maintained a “stable” social distance to students (see Figure 3). T1 starts his lecture by moving his position from the laptop table to the right front of the passageway between students’ desks, and he keeps this kind of social distance to the end of the lead-in. In contrast, T2 (see Figure 4) constantly adjusts her distance with students due to the need to interact. Nevertheless, both teachers’ preference of standing.

#### Posture

Body posture is a modal form that goes hand in hand with gaze and head movement. The form and direction of posture play an essential role in representing and constructing interactive meaning (Norris, 2004). Since the direction of body position coincides with deictic head movement in this study, only the form of body posture is analyzed in terms of the openness of arms. When two arms are hanging in front of the abdomen with hands crossed to form a circular gesture, it is considered a closed or otherwise open posture. T1 and T2 differ significantly in the shift of body posture. T1 presents a closed body posture most of the time. T2, however, switches her form of body posture constantly between open and closed posture, but her open posture takes longer than her closed posture. Different forms of body posture seem to help to build up different teacher images. T1’s closed posture may make students feel solemn, conscientious, and perhaps a little reserved, but he is very humorous during his storytelling. One judge comments that “this contrast makes his storytelling very infectious” [National Advisory Committee on Teaching English to Majors in Higher Education (NACTEMHE), 2016, p. 30]. T2’s shifts between two posture forms indicate that she is a passionate and enthusiastic teacher, but at the same time, she is aware of her teacher identity, as T2 reflects that “I have to posture as what a teacher is expected to be, elegant and scholarly” [National Advisory Committee on Teaching English to Majors in Higher Education (NACTEMHE), 2016, p. 6].

#### Gesture

In this study, any deliberately expressive hand/arm movement that often accompanies spoken language is considered a gesture. When understanding the meaning of gestures, according to McNeill (1992), it is vital to consider them in connection with the accompanying speech. The interconnected relationship between gestures and speech categorizes gestures into four types: iconic gestures, metaphoric gestures, deictic, and beat gestures. Table 6 shows that T1 makes fewer gestural movements than does T2 both in number and duration. As for specific gestural behaviors, T1’s gestures are limited to deictic gestures and beat gestures. His deictic gestures occurred when he raised his hand to switch PPT slides, and beat gestures accompanied spoken language to emphasize a smooth flow of speech. T2, in contrast, makes more frequent use of the four types of gestures, among which deictic gestures are used most frequently, metaphoric gestures the second, ironic gestures the least used, and beat gestures have the most prolonged duration. T2’s frequent use of gestures has much to do with her lead-in strategy, which is explained in the discussion section.

#### Gaze

Eye contact or gaze is a communicative mode subordinate to the mode of spoken language (Norris, 2004, p. 37). The annotation statistics of gaze (see Table 6) show that two teachers resort to eye contact as one of the essential teaching aids to build social contact with students. There are two primary forms of eye contact with students, one with all the students and one with one single student. The former one of the “one-to-many” eye contact shows that the teacher attaches importance to all the students and is treated equally in the teacher’s eyes. T1 utilizes the “one-to-many” type of eye contact all the time during his lead-in, giving students the feeling of “equal treatment.” T2 has direct eye contact with single students during her one-to-one question style of lead-in section, which shows her concern and respect for individual students. However, her “one-to-many” type of eye contact still takes the higher percentage in both numbers and duration. This suggests that the teacher’s one-to-many gaze builds a sense of inclusion conducive to smooth interaction between the teacher and students.

#### Head Movement

In the case of head movement annotated in the study, deictic head movement and head beats are of significance (Norris, 2004, p. 33). Deictic head movement is a modal representation closely related to the shifts of gaze. Head beat is the rapid up and down, or back and forth head movement accompanying spoken language. The annotation result shows (see Table 6) that deictic head movement is the primary representation of head movement, and both teachers shifted their head direction by the shifts of body position or shift of eye contact accompanying spoken language. The head direction shifts are almost in parallel with the frequency of gaze shifts to indicate the direction. This is more often seen in T1’s multimodal pedagogic discourse during his storytelling lead-in.

#### Facial Expression

Teachers’ facial expression is a direct indicator showing their attitudes toward students, which is often categorized into two types: cheerful face and negative face. Teachers often prefer a positive facial expression to evoke warm feelings in students and reflect the teachers’ self-confidence. The annotation result in Table 6 shows that both teachers adopted positive facial expressions more often than negative ones. T1, however, utilized more negative facial expressions (like frowning or a wry smile) in his pedagogic discourse, catering to need when telling the story. That is, T1 utilized more diversified facial expressions to assist in information transmission and thus produce the students’ appealing effect. For example, he frowned when speaking of the protagonist’s work pressure; he gave a wry smile when explaining the stomach ulcer caused by the pressure. An expert judge comments that “the teacher is emphatic with the protagonist, displaying the human side” [National Advisory Committee on Teaching English to Majors in Higher Education (NACTEMHE), 2016, p. 12].

### Multimodal Ensembles of Communicative Modes

The communicative modes are interdependent upon one another in many different ways (Norris, 2004). It is, therefore, of great necessity to find out how the communicative modes are structured to realize the communicative purpose in context. We have found that both teachers’ classroom lead-ins include the same four moves with distinct functions, but they are realized by different specific multimodal ensembles except for the introducing the teaching plan move (see Tables 7 and 8). In the other three moves, T2’s multimodal ensembles are more complex and in scope than T1’s due to their different lead-in strategies. The following is an analysis of how the communicative purpose of each move is realized through the application and coordination of communicative modes.

**Table 7:** T1’s model pattern during lead-in.

S. no.	Moves	Communicative modes pattern
1.	Greeting	Distance + spoken language + gaze + head movement + posture + facial expression
2.	Lead-in activities	Spoken language + facial expression + print (PPT slides) + gaze + head movement + gesture + posture
3.	Introducing the teaching plan	Spoken language + print (PPT slides) + gaze + head movement + gesture + posture + distance + facial expression
4.	Closing the lead-in	Spoken language + posture + print (PPT slides) + gaze + head movement + gesture + facial expression

**Table 8:** T2’s model pattern during lead-in.

S. no.	Moves	Communicative modes pattern
1.	Greeting	Spoken language (proposed feature) + gaze + facial expression + posture + head movement +distance
2.	Introducing the teaching plan	Spoken language + print (PPT slides) + gaze + head movement + gesture + posture + distance + facial expression
3.	Lead-in activities	Spoken language (prosodic feature) + distance + gesture + gaze + head movement + facial expression + print (PPT + whiteboard) + posture
4.	Closing the lead-in	Spoken language (prosodic feature) + posture + print (PPT slides) + gaze + facial expression + gesture + head movement

#### The Interplay of Communicative Modes in Greeting

The greeting is an inseparable part of classroom lead-in, which functions to arouse students’ attention and establish and maintain an interpersonal relationship with students. In the present case of demo class, the contestant teacher meets the students and the judges for the first time, and thus, greeting plays an essential role in affecting the audience’s first impression. To perform this action well, both teachers draw on positive facial expressions, eye contact, social distance, and closed posture with the primary mode of spoken language to establish a friendly and agreeable teacher-students relationship and construct their teacher identity.

However, there are some differences in the salience of specific communicative modes between the two teachers due to their different lead-in strategies. In the case of T1 (seen in Figure 3), there is a noticeable shift of distance to indicate the beginning of class. He steps forward to the students from the laptop desk (as number ① shows) as he is uttering the formulaic expression, “Good afternoon, ladies and gentlemen” with a smiling face and a gaze at all the students. He then chooses to stand in the middle-left front of the students (as number ② shows). He makes his identity clear with the expression “I’m going to be your new teacher” while simultaneously leaning forward and nodding his head with the expression of “for the next 20 min,” highlighting the temporary teacher-student relationship (see the numbers above). During the process, the teacher kept a closed posture, making him look serene and scholarly.

To sum up, in T1’s case, he calls students’ attention to the beginning of the class with shift position and his words, which are most conspicuous in modal intensity. In addition, T1’s gaze, facial expression, and head movement also play a role. Thus, the communicative modes to achieve the purpose of greeting can be structured into the ensemble pattern, as is shown in Table 7.

T2’s modal combination pattern in her greeting action (see Table 8) differs slightly from T1’s. T2 also utilizes the same type of communicative modes but different modal intensities, making her greeting more interactive and constructing an amiable teacher image. Figure 4 shows T2 stands in the middle right front of the students and smiles all the time during the greeting secession. She is aware of the presence of judges and the audience, so she addresses them at the very beginning (see the numbers above). She addresses them “dear students” with an obvious emphasis on “Dear” in her voice when it comes to students.

Meanwhile, she leans forward, looking at the students before her and then sweeping the whole class. This series of nonverbal modal representations have strengthened the referential meaning of “dear students.” The clever wording and the teacher’s nonverbal modes will narrow the distance between the teacher and students. After uttering “How are you today?” the teacher pauses for students’ response with a simultaneous postural shift of leaning forward, encouraging smile and head movement. These chained lower-level actions accompanying T2’s spoken language help convey the communicative meaning of greeting and serve to construct T2’s image as friendly.

#### The Interplay of Communicative Modes in Introducing the Teaching Plan

The primary purpose of introducing the teaching plan is to give students a clear idea about the learning objectives and the procedures to achieve these objectives. T1 and T2 are different in the order of presenting this move. In T1’s demo class, he introduces the teaching plan after the lead-in activity, while T2 follows the regular order of presenting the teaching plan after the greeting move. The two structures have their advantages: T1 states that he “intentionally” puts the teaching plan after the lead-in activity because he “intends to provoke students” curiosity through the storytelling and then create a kind of suspense to students’ [National Advisory Committee on Teaching English to Majors in Higher Education (NACTEMHE), 2016, p. 12]. T2’s order can get students to have a clear idea of what is going to learn and how to learn it from the very beginning to prepare themselves for the following part.

Though different in ordering the move, both teachers assemble communicative modes into the same pattern (See Tables 7 and 8). As is seen in the videos (https://we.sflep.com/Teaching/PastCompetition.aspx?id=6), both teachers mainly rely on spoken language and PPT slides (i.e., print mode) to achieve this purpose. The two modes appear overlapping in that what appears on the PPT screen is precisely the content that the teacher speaks. In addition, both teachers employ deictic movements comprised of postural shifts, head movements, gestures, and gazes to indicate a shift of attention to the PPT screen. For example, T1 and T2 shifted their posture and gaze to turn the students’ attention to the PPT slide. These deictic movements coordinate with and assist spoken language to convey information, promote a sense of transition and add cohesion to the representation of interactional meaning.

#### The Interplay of Communicative Modes in Organizing Lead-In Activity

The move of presenting a lead-in activity is the critical part of lead-in, whose purpose is to acquaint students with the background information of the topic and prepare them for the following class procedure. In these two cases, both teachers succeed in attracting students’ attention and arousing their enthusiasm and initiative in learning, which can be shown from the students’ responses in class. Spoken language still takes up high modal density, and the interactional meaning is realized by spoken language and complexity of other communicative modes, like print, proxemics, gaze, posture, gesture, layout, head movement, etc.

However, there are differences in modal selection and combination because the two teachers adopted different lead-in methods. T1 adopts the storytelling way to tell students how Rick decides to quit his job and become a housefather. It seems dull that T1 himself speaks all the time without involving students’ participation. However, he is a good storyteller by appealing to students with clear and interactive language and multiple other communicative modes, especially facial expressions, gaze, and head movement. In T1’s storytelling style of lead-in, he sets up suspense and questions to attract students’ attention to better follow him to know the background information of the topic. For example, he starts with “Today is not about me. Today is about a guy whose name is Rick.,” and then he pops up the question “Who is Rick?” on the PPT screen, which arouses students’ curiosity to find out more information about Rick. He also uses the expressions like “let me tell you this is a stressful job” “sounds perfect, right?” to make his narration dialogue directly appealing to students.

His narration is more appealing because he utilizes various communicative modes, such as facial expressions, PPT images, posture, head movements, gaze, layout, and gesture, to coordinate with spoken language. The complexity of communicative modes supplements and strengthens the information conveyed through spoken language and made the narration vivid and appealing. For example, when talking about Rick suffering from a gastric ulcer due to tremendous work pressure, the teacher’s head is tilted, frowning, and his face is solemn, showing a sad expression. He smiles broadly when he tells the students that it was only a joke about Rick’s death obituary. The teacher’s multimodal combination made his introduction so appealing that students’ enthusiasm is aroused for the upcoming discussion. Just as one expert judge commented on his lead-in, “The teacher’s natural description produces a great affinity, which makes the interaction in the following section between teachers and students is natural” [National Advisory Committee on Teaching English to Majors in Higher Education (NACTEMHE), 2016, p. 24].

However, T2’s modal combination pattern is quite different from T1’s due to the question-and-answer approach adopted to familiarize students with the topic. T2’s procedures of presenting lead-in activity are pretty straightforward. She first brings up technology with the PPT screen displaying the technology products in the contemporary world. She then interviews students for reasons of using a cell phone. After that, she asks all the students to brainstorm what they have lost if using cellphone too much and writes critical points on the whiteboard. She points out that students have expressed their point of view on technology, but the class focuses on exploring the author’s view. Therefore, students will be intrigued to delve into the author’s view.

During the whole process, T2 utilizes a multiplicity of communicative modes to construct this communicative purpose (see Table 8). Due to the high interactive feature, T2’s ensemble of communicative modes is different from T1. First of all, her spoken language takes on apparent prosodic features, such as pause and high pitch. For example, the teacher pauses after asking a question and observes the student’s reaction. She raises her voice in surprise when one student provides an unexpected answer to her question. While listening to students answering questions, she encourages them to speak more through eye contact, smiling facial expressions, and nodding head movement. Secondly, in the process of interaction, the teacher constantly adjusts the distance from students. She will approach the students and shorten the distance when asking questions or walking away to write on a whiteboard when listening to students answering questions. Thirdly, the teacher utilized a more diversified form of the mode of print. Besides PPT images, the teacher also writes on the whiteboard to directly presents students’ views on a cellphone, which makes up for the shortcomings of PPT images.

In terms of model density and complexity of communication modes, the modal representation forms adopted by T2 are more apparent and more distinguishable, such as walking in the classroom and writing on the whiteboard, and the modal forms of spoken language, body posture, and head movements are more prominent as well. All in all, the modal form adopted by T2 has something to do with the lead-in method she adopted.

#### The Interplay of Communicative Modes in Ending the Lead-In

The move of ending the lead-in is the manner to direct students to the following procedure of the class. Both teachers utilize spoken language accompanied by deictic movements to indicate the end of lead-in and shift students’ focus to another new higher-level action. Moreover, the PPT slide also plays a significant role in informing the students of the following teaching procedure. That is, they take on modal intensity in the meaning-making process. However, there is a slight difference between the two teachers in applying and combining communicative modes.

As is shown in videos (https://we.sflep.com/Teaching/PastCompetition.aspx?id=6), when T1 announces, “Let us proceed to the first part” – a sign of ending the lead-in – he utilizes deictic movements, such as body posture, gesture, head movement, and gaze to direct students’ attention to the next part of the class. At the same time, the PPT slide shows the information of the following teaching procedure, complementary with T1’s verbal information. T2 still adopts a more interactive way to inform the students of the end of lead-in, and she employed a more complicated combination of communicative modes. She uses complex modal forms, such as proxemics, body posture, gestures, head movements, facial expressions, and gaze to assist spoken language in meaning delivery. For example, she tags with “all right” after she tells the students that they will read something to explore the author’s view. At the same time, she leans forward with a gentle smile and an expectant gazing at the students, calling for their response. When they respond positively, the teacher thanks the students and turns around to change the PPT slide, indicating they are moving on to the following teaching procedure.

## Discussion

The central issue of this study was how EFL teachers engaged students during classroom lead-in employing multimodal pedagogic discourse, which is a reflection of teachers’ multimodal competence. We probed into this question from two aspects: the teachers’ choice of communicative modes and the way they constructed multimodal ensembles to realize the functions of classroom lead-in. Our findings corroborate the previous point of view that classroom teaching is a multimodal experience that happens through orchestration of spoken language and an array of other communicative modes, such as gesture, gaze, and facial expression (Kress et al., 2005; Jewitt, 2008; Peng, 2019; Lim, 2021). Our findings also reveal that the two highly-qualified EFL teachers possess the multimodal competence to construct multimodal pedagogic discourse during the classroom lead-in. And their multimodal competence enables them to choose and assemble communicative modes to realize the functions of classroom lead-in: gaining attention, stimulating motivation, setting up teaching objectives, building and establishing communicative links.

The teacher is recognized as “a designer of the learning experience of students” (Mercer and Dörnyei, 2020, p. vi). Teacher’s multimodal competence “plays a crucial role in integrative lecturing, especially when the language of communication is other than one’s own” (Morell’s, 2018, p. 70). Our study supports Morell et al. (2020) in that multimodal ensembles indeed foster classroom engagement. In addition, it verifies Lim’s (2017, p. 26) claim that teachers’ orchestration of multimodal recourses encourages a “more congruent and effective” learning experience for students. During the classroom lead-ins, both teachers’ orchestration of communicative modes was utilized to realize the pedagogic functions in the four moves. This is a demonstration of their high awareness of multimodal competence. Our findings show that EFL teachers’ multimodal competence in performing a multimodal pedagogic discourse during lead-in allows stranger students to follow them and establish a communicative link step by step. Education is relational, and a close, caring teacher-student relationship plays a vital role in students’ classroom engagement, learning, and performance (Furrer et al., 2014; Mercer and Dörnyei, 2020). In this way, students are aware of the teaching plan, acquainted with the topic theme, and motivated for the following procedures of class activities. van Lier (1996, p. 112) points out that language teaching is most effective when the teacher “stimulates intrinsic motivation, to take advantage of natural interests, curiosity, and emergent rewards.”

Student classroom engagement is often considered a good predictor of student learning and development (Pike et al., 2012). Among the different dimensions for the realization of engagements, Mercer and Dörnyei (2020, p. 3) perceive “engagement to always be associated with an action.” Behavioral participation is the most attractive predictor for students’ participation in the classroom (Fuller and Marler, 2009). Lim (2021, p. 2) observes that “[w]hether the students feel safe to participate or are inhibited from speaking up are often a result of the meanings they perceive from their teachers’ embodied semiosis.” Therefore, in order to engage students to participate in the classroom, two highly-qualified EFL teachers preferred to choose embodied modes like positive facial expressions, “one-to-all gaze and interpersonal distance” to build an intimate personal relationship with “stranger” students and make them feel a sense of belonging to the class not just as “onlookers” of the class. Cemalcilar (2010) notices that students who are more likely to feel a sense of belonging will be more engaged in school work.

It is worth noting that the two EFL teachers possess high-level multimodal competence, which enables them to choose and assemble a multiplicity of communicative modes under the different lead-in strategies they adopted. T1’s storytelling lead-in appears to involve fewer students’ behavioral participation as there was no question-answer section during the storytelling. However, it impacted students’ emotional/affective engagement – it is an internal dimension of engagement, referring to learners’ interests and sense of belonging/attachment (Fredricks et al., 2004). Judged from T1’s multimodal ensembles, it is noted that T1’s facial expression, gaze, PPT slides accompanied his clever choice of spoken language weighted high in modal density. T1 orchestrated these semiotic resources to design a learning experience for students by arousing students’ interest in the topic. Jewitt (2008, p. 262) observes that “the way teachers use multimodal semiotic resources like gaze, body posture, and space in the classroom affects literacy.”

In contrast, T2’s question-answer lead-in involves more students’ behavioral engagement in that students are expected to participate in activities designed by the teacher. Behavioral engagement draws on the idea of participation (Fredricks et al., 2004, p. 60). The classroom setting refers to the students’ active and participatory involvement in academic activities (Mercer and Dörnyei, 2020). That is to say, students’ behavioral engagement can be observed from the actions or performance in terms of active participation in interactive classroom activities (Fredricks et al., 2004). Seen from T2’s multimodal ensembles, it is found that her pattern is mainly different from T1’s. Communicative modes like the teacher’s positioning, gesture, movement, and gaze take on more shifts and higher modal density because these modes have much to do with provoking students’ behavioral engagement. Among all the dimensions of engagement, behavioral participation in the classroom is considered the “core construct, most prototypical of engagement” (Skinner, Furrer, Marchand, and Kindermann, 2008, p. 778; Mercer and Dörnyei, 2020, p. 3). Therefore, it is crucial for teachers’ to use their multimodal competence to engage students to participate actively to achieve positive academic outcomes.

It is also noted that teachers’ multimodal competence has much to do with the different engagement they intend to provoke in students. T2’s question-and-answer lead-in involves students’ behavioral participation. In order to engage students, T2 used an interactive tone of voice, constant gaze shifts, frequent shifts of positioning distance with students, and diversified gestural forms. Peng (2019) observes that teachers’ gestures and spatial positions predict students’ willingness to participate in the classroom. This is also the case in T2’s multimodal ensembles. T2’s complex multimodal ensembles during the classroom lead-in show that T2 provides necessary support to students, and she is also very passionate about what she is to do. If understood in terms of the principles suggested by Mercer and Dörnyei (2020), T2’s multimodal competence displayed during the lead-in helps to facilitate a more behavioral engagement.

Compared with T2’s complex multimodal ensembles, T1 seems to be more “simplistic” in telling students a story with his words accompanied with embodied actions like eye contact, facial expressions, and information on the PPT slides. However, T1 also fully engaged students and laid a solid foundation for the formal presentation of the topic in the following section. We think the reasons might be found in another principle that Mercer and Dörnyei (2020) suggested facilitates the more compelling aspects of engagement. First of all, T1 is physically approachable when he chooses a position to tell students the story. More importantly, T1’s approachability is reflected through his humor. Mercer and Dörnyei (2020, p. 54) hold that humor can be another way to “lower the affective filter and generate positive affect,” revealing to learners the “human” side of the teacher. Wanzer et al. (2010) explain that humor might lead to deeper cognitive processing, better relationships, and more effective learning when the form of humor is appropriate. Secondly, the teacher is emphatic. Empathy means being able to step into somebody else’s shoes and see the world from their perspective (Mercer and Dörnyei (2020, p. 55). This can be seen from T1’s shift of facial expressions accompanying storytelling and his clever choice of linguistic symbols where he used Chinese symbols to explain abstract English words, facilitating students’ understanding.

Finally, the teacher’s multimodal competence also helps to shape a teacher’s image and teaching style. Norris (2004, p. 137) argues, “Every higher-level action that a social actor engages in constructs the person’s social world.” Our findings also suggest that both teachers construct their professional image through the appropriate application of multimodal communicative modes reflected in their words and actions. T1 impresses the students and judges with his calm and steady image, which is constructed by his application of closed posture, stable distance with students, and infrequent shifts of gestures. Meanwhile, he also is regarded as humorous as his skillful and humorous style of telling the story fully engaged the students during the lead-in. T2, on the other hand, builds up her personal charisma as a passionate and approachable teacher to students through her frequent application of nonverbal communicative modes to complement and reinforce her spoken language.

## Conclusion

This study has explored the multimodal pedagogical discourse of classroom lead-ins delivered by two highly-qualified EFL teachers during a national teaching competition. The findings reveal that language teachers’ high-level multimodal competence play a positive role in engaging students during the classroom lead-in. The multimodal competence enables them to choose and assemble a multiplicity of communicative modes along with the primary mode of spoken language depending on the communicative purpose in context. In addition, the multimodal pedagogic discourse they produced is largely in accordance with the different lead-in strategies they adopted.

However, this multimodal interaction analysis only focuses on a very small corpus of two EFL teachers’ classroom lead-ins, and thus, we can only reach some tentative findings. More future research with larger datasets is expected to verify our findings. In addition, some research-based and pedagogical implications might be drawn from the present research. In the case of multimodal pedagogic discourse study, as Kress et al. (2005) have suggested that multimodal analysis of pedagogic discourse contributes to a more complete understanding of the teaching and learning that occur in the classroom, future studies are suggested to take into account the overall language teaching process, so that we will better understand the degree of teachers’ multimodal competence contributes to effective EFL teaching. Particularly, more empirical research is expected to investigate the relations between the teacher’s multimodal competence and students’ classroom engagement as well as the effects on students’ academic performance.

In terms of pedagogic implication, EFL teachers need to be aware of the significance of multimodal competence and learn to put it into practice to engage students in the classroom setting. For the first thing, language teachers should take care of teacher talk. Mercer and Dörnyei (2020) suggest that teacher talk in the language classroom has the power to affect not only language learning but also the teacher-student relationship. During the teaching plan period, language teachers are advised to prepare in advance what to say and how to say it to positively engage students. Secondly, language teachers need to be aware of the significance of nonverbal communicative modes accompanied by language, as they can also “talk” in the meaning-making process and learn to combine them according to modal density into multimodal ensembles. Thirdly, when teachers make modal combinations, they need to consider the complexity of the lead-in strategies, as different lead-in strategies involve different multimodal pedagogic discourse. Therefore, this study suggests that more future research could be conducted on this topic to suggest the relations between multimodal competence and students’ classroom engagement.

## Data Availability Statement

The original contributions presented in the study are included in the article/supplementary material, further inquiries can be directed to the corresponding author.

## Ethics Statement

The studies involving human participants were reviewed and approved by the institutional review board of Foreign Language School, Jiangsu University of Science & Technology. The patients/participants provided their written informed consent to participate in this study.

## Author Contributions

YQ and PW shared equal contributions: They conceived of the presented idea, collected, and analyzed data. YQ wrote the manuscript. PW revised the manuscript. All authors contributed to the article and approved the submitted version.

## Funding

This research was supported by Social Science Fund of Jiangsu Province (Grant No. 21YYB011), Jiangsu Provincial Department of Education (Grant No. 2020SJA2095) and Xi’an International Studies University (Grant No. BSZA2019002).

## Conflict of Interest

The authors declare that the research was conducted in the absence of any commercial or financial relationships that could be construed as a potential conflict of interest.

## Publisher’s Note

All claims expressed in this article are solely those of the authors and do not necessarily represent those of their affiliated organizations, or those of the publisher, the editors and the reviewers. Any product that may be evaluated in this article, or claim that may be made by its manufacturer, is not guaranteed or endorsed by the publisher.
